# Supplementary material for: Telomere shortening and the transition to family caregiving in the Reasons for Geographic and Racial Differences in Stroke (REGARDS) study
Source: PLoS One. 2022 Jun 3;17(6):e0268689. doi: 10.1371/journal.pone.0268689 (PMC9165822; doi:10.1371/journal.pone.0268689)
Supplement: S1 Table — (DOCX) [file pone.0268689.s001.docx]

**S1 Table**. Telomere Research Network Reporting Guidelines.

| **ITEM** | **DESCRIPTION** |
| --- | --- |
| **Sample Type, Storage, Extraction, and Integrity** | |
| Sample type | DNA samples were extracted from packed cells from EDTA tubes. |
| Sample storage conditions, including temperature, duration, and buffer | Packed cells were stored at -80^○^C until DNA was extracted in October/November 2018. |
| DNA extraction method | DNA was extracted using the Gentra Puregene DNA extraction kit (Qiagen) using manufacturers guidelines. |
| DNA storage conditions, including freeze-thaw cycles | Extracted DNA was stored at -80°C in manufacturers buffer until shipped frozen on dry ice to Pennsylvania State University (University Park) in April 2019. On average, there was a minimum of 3 freeze thaws for DNA samples between extraction and the qPCR assay. Once received at Penn State, DNA samples were stored at -80 until telomere length analysis was conducted. DNA concentration was performed using Quant-iT PicoGreen dsDNA assay kit (Thermofisher). DNA dilutions for the telomere length were done immediately once the picogreen analysis was determined and stored at 4°C. The telomere length analysis was performed immediately upon diluting the DNA to the correct concentration. Samples needing to be reassessed for qPCR assays (n=18) were diluted one additional time (when needed). DNA samples were stored for an average of 6 months between extraction and the PicoGreen assay, and for another 6 months between the PicoGreen assay and qPCR assay. |
| Method of documenting DNA quality and integrity | DNA was quantified for all samples using Quant-iT PicoGreen (mean= 5.3 ng/uL). DNA purity and quality were assessed using a Nanodrop to measure the 260/230 and 260/280 ratios. No exclusionary criteria were imposed prior to assays. |
| Percentage of samples specifically tested for DNA quality and integrity | 100% |
| **qPCR Assay** | |
| Method (qPCR, MMqPCR, aTL, etc.) | Each qPCR telomere length assay was comprised of two qPCR runs, one run quantifying telomere content (T) and a second run quantifying genome copy number (S) using the single copy gene *IFNB1.* The two runs (T & S) were always performed on the same day using the same DNA aliquot which was stored at 4°C between runs (~2.5 hours). Each qPCR run hosted triplicate reactions of 23 samples, 6 standards, 4 positive controls and one no template control on 100 well disks. A total of 94 qPCR assays were performed across a period of 19 weeks for analysis of all samples. |
| PCR machine type | Qiagen Rotor-Gene Q using 100 well disks |
| Source of master mix and reagents, and final reaction volume | The final master mix for the telomeric and *IFNB1* qPCR amplification contains 1x QuantiTect SYBR Green Master Mix (Qiagen), 0.1U Uracil Glycosylase (Thermo Fisher Scientific), 0.1 uM forward primer, 0.1 uM reverse primer, and 3 ng DNA in a 20 uL reaction. |
| Telomere primer sequences and concentration | Forward Primer: 5'CGGTTTGTTTGGGTTTGGGTTTGGGTTTGGGTTTGGGTT3′  Reverse Primer: 5'GGCTTGCCTTACCCTTACCCTTACCCTTACCCTTACCCT3′  All primers were HPLC purified and lab ready to dilute to a 10uM working concentration |
| Single copy gene name, primer sequences, and concentration | *IFNB1* Forward Primer 5' GGACTGGACAATTGCTTCAAG 3’  *IFNB1* Reverse Primer 5' CCTTTCATATGCAGTACATTAG 3′  All primers were HPLC purified and lab ready to dilute to a 10uM working concentration |
| Full PCR program description including temperature, times, and cycle numbers | HOLD: 50°C – 2min (UNG activation)  HOLD: 95°C – 15min (Taq Hot Start activation)  45 CYCLES: 95°C – 15s followed by 60°C for 1 min (data acquisition)  MELT CURVE: Ramp from 50°C to 99°C rising by 1°C each step |
| PCR efficiency of single copy gene and telomere primers | Telo: R^2^ = 0.999 (range 0.997 – 0.999); Efficiency=0.972 (range 0.82 – 1.06)  *IFNB1*: R^2^ =0.997 (range 0.990 – 0.999); Efficiency=0.90 (range 0.84 – 1.01) |
| Source and concentration of control samples and standard curve | 3 positive controls were randomly selected from within the sample to control for variation across T and S runs. A known length DNA from a human cell line (1301: T cell leukemia; Sigma cat# 01051619) acted as a calibrator for the Ct value and was purchased from Sigma. Standards consisted of double stranded oligomers using a ten-fold dilution of the highest concentration being 60 pg for the telomeric qPCR and 2 pg for the IFNB1 qPCR. |
| **Data Analysis** | |
| Mean and standard deviation or median range of telomere lengths | T/S ratio mean (SD) = 6.05 (1.06) |
| Number of sample replicates | Each sample was assessed for T and S on a single run with three replicates within the run. If the sample did not pass quality control criteria described below it was run a second time. |
| Level of independence of replicates | Replicates were drawn from the same DNA aliquot (i.e., the same tube). |
| Analytic method, considering replicate measurements, to determine final length | T and S estimates were calculated using the Ct_Telo/IFNB1_ values for individual replicates. T/S ratios were calculated using the average Ct_Telo_ and average Ct_IFNB1_ across replicates. |
| Method of accounting for variation between replicates | When the standard deviation across replicate Ct values was greater than 0.10, replicate Ct values were evaluated based upon their deviation from mean Ct across triplicates. If one replicate deviated from the mean Ct by more than 15%, it was considered an outlier and the mean Ct was recalculated using two replicates. An average of 5 T replicates and 7 S replicates were dropped per run (*in this case T/S ratios were calculated using the average across duplicate Ct values*). In the case where Ct_TELO.IFNB1_ standard deviation across replicates was still greater than 0.10 after removal of a single outlier or was greater than 0.10 without a clear outlier defined by the criteria above, the sample was reassessed for both telomere content and genome copy number and subjected to the same quality control evaluation. A total of 18 samples were rerun a second time and a total of 12 samples were rerun a third time. |
| Method of accounting for well position effects within plates | The unique rotary design of the Rotor Gene Q is optimized to minimize well position effects. As such no accounting for well position effects was performed. |
| Method of accounting for between plate effects | To control for inter-assay variability, five controls samples were run on each plate. For each plate, the Ct value of each control DNA was divided by the average Ct value for the same DNA across all runs to get a normalizing factor for that sample on a given plate. This was done for all controls to get an average normalizing factor for that plate. In this manner, the mean (±SD) intra-assay CV across all samples was less than 1.73% (+0.01) and the mean (±SD) inter-assay CV was 2.85% (+0.02). |
| % of samples repeated and % of samples failing QC and excluding from further analyses | 18/1035 = 1.7% of samples repeated.  12/1035 = 1.2% of samples failed QC and excluded from analyses. |
| Acceptable range of PCR efficiency for single copy gene and telomere primers | TELO: 0.82 – 1.06 (*larger window because the primer pair does not perfectly match telomere repeats;* 0 plates outside this range)  IFNB1: 0.84 – 1.01 (0 plates outside this range) |
| ICCs of samples/study groups to address variability | ICC not calculated. |
| T/S ratio transformed to a z-score prior before comparison across methods/studies | N/A. No comparison across studies was conducted. |
| How samples nested within families were accounted for | qPCR assays were performed in a blinded fashion to familial status (if applicable). However, longitudinal samples from the same individual were nested together and run on the same plate. |
