# Supplementary material for: Telomere shortening and the transition to family caregiving in the Reasons for Geographic and Racial Differences in Stroke (REGARDS) study
Source: PLoS One. 2022 Jun 3;17(6):e0268689. doi: 10.1371/journal.pone.0268689 (PMC9165822; doi:10.1371/journal.pone.0268689)
Supplement: S2 Table — (DOCX) [file pone.0268689.s002.docx]

S2 Table. Bivariate correlations between measures of telomere length, age, and perceived stress.

| **Variable 1** | **Variable 2** | **DF** | **r** | **P** |
| --- | --- | --- | --- | --- |
| ΔT/S | Visit 1 ln(T/S) | 411 | -0.483 | <0.001 |
| ΔT/S | Visit 2 ln(T/S) | 411 | 0.458 | <0.001 |
| Visit 1 ln(T/S) | Visit 2 ln(T/S) | 411 | 0.557 | <0.001 |
| ΔT/S | Age, Visit 1 | 411 | -0.042 | 0.390 |
| ΔT/S | Age, Visit 2 | 411 | -0.050 | 0.313 |
| Visit 1 ln(T/S) | Age, Visit 1 | 411 | 0.058 | 0.237 |
| Visit 2 ln(T/S) | Age, Visit 2 | 411 | 0.021 | 0.676 |
| ΔT/S | PSS, Visit 2 | 405 | -0.012 | 0.815 |
| Visit 1 ln(T/S) | PSS, Visit 2 | 405 | -0.014 | 0.775 |
| Visit 2 ln(T/S) | PSS, Visit 2 | 405 | -0.025 | 0.609 |
| Age, Visit 1 | PSS, Visit 2 | 405 | 0.030 | 0.540 |
| Age, Visit 2 | PSS, Visit 2 | 405 | 0.014 | 0.775 |
| **Abbreviations**: DF-degrees of freedom, r- correlation coefficient, PSS- Perceived Stress Scale | | | | |
