# Supplementary material for: Telomere shortening and the transition to family caregiving in the Reasons for Geographic and Racial Differences in Stroke (REGARDS) study
Source: PLoS One. 2022 Jun 3;17(6):e0268689. doi: 10.1371/journal.pone.0268689 (PMC9165822; doi:10.1371/journal.pone.0268689)
Supplement: S3 Table — (DOCX) [file pone.0268689.s003.docx]

S3 Table. The change in telomere length (ΔT/S) across perceived stress scale categories.

| **PSS Category Comparison^a^** | **CTS Telomere Study (N=407)** | | **CTS Caregivers Only (N=202)** | |  |
| --- | --- | --- | --- | --- | --- |
|  | **ΔT/S Mean Difference** | **95% Confidence Limits** | **ΔT/S Mean Difference** | **95% Confidence Limits** |  |
| Low-Moderate | 0.024 | -0.237, 0.284 | 0.147 | -0.222, 0.516 |  |
| Low-High | 0.035 | -0.254, 0.325 | 0.224 | -0.157, 0.606 |  |
| Moderate-Low | -0.024 | -0.284, 0.237 | -0.147 | -0.516, 0.222 |  |
| Moderate-High | 0.012 | -0.286, 0.310 | 0.078 | -0.301, 0.457 |  |
| High-Low | -0.035 | -0.325, 0.254 | -0.225 | -0.606, 0.157 |  |
| High-Moderate | -0.012 | -0.310, 0.286 | -0.078 | -0.457, 0.301 |  |
| **^a^**Low category=0-1; moderate category=2-4; high category=5+  **Abbreviations**: PSS- Perceived Stress Scale | | | | | |
