# Supplementary material for: Telomere shortening and the transition to family caregiving in the Reasons for Geographic and Racial Differences in Stroke (REGARDS) study
Source: PLoS One. 2022 Jun 3;17(6):e0268689. doi: 10.1371/journal.pone.0268689 (PMC9165822; doi:10.1371/journal.pone.0268689)
Supplement: S1 Fig — Left panel: ln(T/S) at baseline (visit 1) and right panel: the change in ln(T/S) over the follow-up period. (DOCX) [file pone.0268689.s004.docx]

**
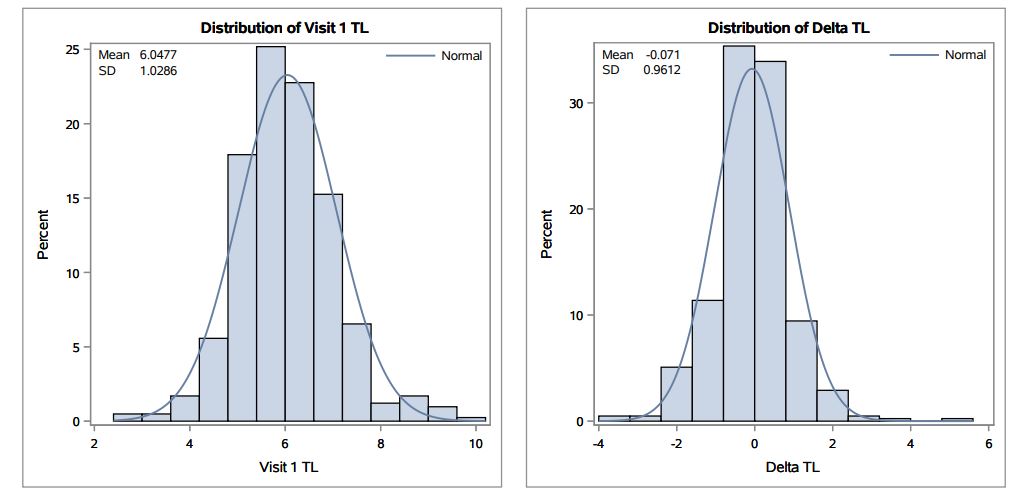
**

**S1 Figure.** The distribution of telomere length. Left panel: ln(T/S) at baseline (visit 1) and right panel: the change in ln(T/S) over the follow-up period.
