# Supplementary material for: Telomere shortening and the transition to family caregiving in the Reasons for Geographic and Racial Differences in Stroke (REGARDS) study
Source: PLoS One. 2022 Jun 3;17(6):e0268689. doi: 10.1371/journal.pone.0268689 (PMC9165822; doi:10.1371/journal.pone.0268689)
Supplement: Attachment — Submitted filename: Response to Reviewers.docx [file pone.0268689.s005.docx]

**PONE-D-22-03368**

**Telomere shortening and the transition to family caregiving in the Reasons for Geographic and Racial Differences in Stroke (REGARDS) study.**

The authors would like to thank the journal, editor, and reviewer for their very insightful and constructive comments of our manuscript. We have responded to all the comments below. The original comment/review is in regular black font. Our response is in red font. Any wording that refers to changes in the manuscript (e.g., page/line numbers or direct quotes) are in red font with yellow highlighting. We believe that the manuscript is improved and hope it is now suitable for publication in *PLOS ONE.*

**Journal Requirements:**

When submitting your revision, we need you to address these additional requirements.

1. Please ensure that your manuscript meets PLOS ONE's style requirements, including those for file naming.

- The authors thank the journal for providing the style templates. We apologize for overlooking these style requirements and for having inconsistent font and sizes. We have addressed the following formatting changes on the title page and within the manuscript:
- Title page- We corrected the article title by writing it in sentence case versus title case (page 1, lines 4-6).
- Main body- We corrected the manuscript by following the guidelines and ensuring the correct font size per each header, sentence case, and consistent Times New Roman typeface.

2. Thank you for stating the following in the Acknowledgments Section of your manuscript: 
[This work was supported by the cooperative agreement U01 NS041588 co-funded by the National Institute of Neurological Disorders and Stroke (NINDS) and the National Institute on Aging (NIA), National Institutes of Health, and Department of Health and Human Service. The Caregiving Transitions Study was further supported by an investigator-initiated grant (RF1 AG050609) from the NIA. Additional support was provided by the Johns Hopkins University Claude D. Pepper Older Americans Independence Center funded by the NIA (P30 AG021334). The content is solely the responsibility of the authors and does not necessarily represent the official views of the NINDS or the NIA.  Representatives of the NINDS were involved in the review of the manuscript but were not directly involved in the collection, management, analysis, or interpretation of the data. The authors thank the other investigators, the staff, and the participants of the REGARDS study for their valuable contributions.  A full list of participating REGARDS investigators and institutions can be found at <http://www.regardsstudy.org>.]

- The authors have made the following edits in the manuscript. The acknowledgements section should read as follows: “The authors thank the other investigators, the staff, and the participants of the REGARDS study for their valuable contributions.  A full list of participating REGARDS investigators and institutions can be found at <http://www.regardsstudy.org>.”

We note that you have provided funding information that is not currently declared in your Funding Statement. However, funding information should not appear in the Acknowledgments section or other areas of your manuscript. We will only publish funding information present in the Funding Statement section of the online submission form. 

Please remove any funding-related text from the manuscript and let us know how you would like to update your Funding Statement. Currently, your Funding Statement reads as follows: 
 [This work was supported by the cooperative agreement U01 NS041588 co-funded by the National Institute of Neurological Disorders and Stroke (NINDS) and the National Institute on Aging (NIA), National Institutes of Health, and Department of Health and Human Service. The Caregiving Transitions Study was further supported by an investigator-initiated grant (RF1 AG050609) from the NIA. Additional support was provided by the Johns Hopkins University Claude D. Pepper Older Americans Independence Center funded by the NIA (P30 AG021334). The content is solely the responsibility of the authors and does not necessarily represent the official views of the NINDS or the NIA.  Representatives of the NINDS were involved in the review of the manuscript but were not directly involved in the collection, management, analysis, or interpretation of the data.]

- We have amended this funding statement slightly to include the following line, “RAM receives support as the Sarah Miller Coulson Scholar in the Johns Hopkins Center for Innovative Medicine”. We apologize for any confusion and inconvenience and appreciate you changing the online submission form on our behalf. This amended statement is also addressed within our cover letter.

3. We note that you have stated that you will provide repository information for your data at acceptance. Should your manuscript be accepted for publication, we will hold it until you provide the relevant accession numbers or DOIs necessary to access your data. If you wish to make changes to your Data Availability statement, please describe these changes in your cover letter and we will update your Data Availability statement to reflect the information you provide.

- We would like to change the data availability statement to state: “In cooperation with the Institutional Review Board of The University of Alabama at Birmingham, the REGARDS project facilitates data sharing through formal data use agreements. Investigators who wish to access the data and code for these analyses should send their requests to [regardsadmin@uab.edu](mailto:regardsadmin@uab.edu).” This has been described in the cover letter as well. The authors appreciate the assistance and apologize for any inconvenience.

**Additional Editor Comments:**

For Fig. 1, you may move to supplemental data.

- Thank you for this comment. Upon the suggestion from the reviewer, we have deleted Figure 1 altogether, as the information was already present in the supplemental table 2.

**Reviewers' comments:**

- The authors want to thank the reviewer for their insightful and constructive comments. We apologize for the inconsistent font and errors in the original manuscript. We have corrected the errors, performed a spelling and grammar check, and reformatted according to the PLOS ONE template.

**Particularly the introduction and the methods section could use a spelling and grammar check. And also, please make sure that you use the same font throughout the whole manuscript. See more detailed queries below:**

- The authors thank the reviewer for their comment. We have reworked the introduction section and have thoroughly performed a spell and grammar check. We have also made sure that the font throughout the entire manuscript is Times New Roman 12pt font, with the exceptions of 10 pt. in the Table footnotes, and the appropriate size font for each header/sub-header as defined by the PLOS ONE style template.

**Introduction**
Please do not use citations from other papers, such as: "The authors stated that “Women with the highest levels of perceived stress have telomeres shorter on average by the equivalent of at least one decade of additional aging compared to low stress women" Please delete and paraphrase.

- The authors thank the reviewer for the comment regarding direct quotations from other papers. We have deleted this quotation, as well as others throughout the manuscript. The new literature review of previous TL and caregiving studies are described in lines 88-111 on pages 4-5.

Why is the following relevant for this manuscript: "This publication has been cited over 3,000 times as of December 2021 according to Google Scholar, and has been widely shared on both family caregiver websites such as the Caregiver Action Network (25], and in foundation and news reports on family caregiving[26, 27]." Please delete.

- The authors have deleted this line as well. We have paraphrased on line 90-93 (page 4): “Three earlier cross-sectional studies have reported a relationship between shorter TL and being a caregiver [12, 26], or shorter TL and longer duration of caregiving and/or higher perceived stress among caregivers [27].”

In general in the introduction the authors provide too much details of every referred study. These methodological details would only be relevant if you are going to do it differently or that these methodological details explain why they ended up with certain results. Please focus only on the details that are relevant. I really miss a logical summary of the preceding literature on a more meta-level; I am not interested in all details, but I want to know what the results of preceding studies mean and to which extent they are comparable and if not why not and then – what it is that the current study will add to existing knowledge.

- The authors thank the reviewer for their comment aiming at focusing the literature review on the introduction. We have focused on the broader implications of the previous telomere and/or caregiving literature. Please refer to lines 88-111 on pages 4-5.

The aim of the current study seems overstated “These findings could address previous conflicting findings on this issue which is of great importance not only for our scientific understanding of the relationship between stress, telomeres, and health, but also for guiding our understanding of the pressing public policy and clinical issues faced as our aging population depends increasingly on family caregivers to provide care”. How so will you look at health and clinical issues related to aging? You will only look at telomere length and not something that is more clinically relevant. Moreover, as you show in the introduction this is hardly the first study to look at the relation between caregiving and telomere length.

- The authors thank the reviewer for these comments. We have rephrased the aim of the current study as to not overstate the findings (lines 122-131 on pages 5-6: “This design enabled examination of whether the transition to incident caregiving was associated with greater changes in TL. We further examined whether perceived caregiving stress was associated with TL within the caregiving case group, and finally whether TL was associated with a more general measure of perceived stress in the full CTS. We hypothesized that the transition to caregiving would constitute a form of chronic stress and that the transition to caregiving would be accompanied by a reduction in TL. The rich data resource paired with banked samples in REGARDS allowed the current study to address limitations of previous research yielding conflicting findings on the relationship of caregiving stress and TL changes.”
- We have inserted a phrase stating that while there are several studies preceding this one that examined the relationship between telomere length and caregiving, we have not been able to identify one where the focus has been on the transition to caregiving (and the stress/life changes within) and the change in telomere length. Please see lines 116-118 on page 5: “To the best of our knowledge, there are no previous studies that have examined how the caregiving transition (compared to non-caregiving) influences prospective changes in TL within the same individuals compared with non-caregiving.”
- We have removed the phrasing implicating any direct clinical relevance, as this manuscript does not focus on other biomarkers, such as inflammatory biomarkers, just telomere length.

Also please make clear whether you think these associations between caregiving and telomere length are specifically related to caregiving, or that caregiving is a form of chronic stress? And would this be the case for all types of caregiving, or not? In relation to stress (chronic stress, trauma, life events) several longitudinal studies with much longer follow-up and more assessments of telomere length have already been published (see for instance Gerritsen et al in Psychosomatic Medicine).

- We studied TL as a possible biomarker for stress-related cellular aging and decreased longevity. Therefore, we hypothesized that the transition to caregiving would constitute a form of chronic stress, consistent with the common perspectives in the family caregiving literature.
- In an added secondary analysis, we show that chronic stress and caregiving status are associated, but, similarly to our primary analysis, perceived stress and TL were not. Please refer to the new data in Table 1 and Supplemental Table S3. However, it is important to note that the individuals from the CTS are from an epidemiological sample and not a clinical sample and both caregivers and controls tend to not be overly stressed using the Cohen 4-item stress scale, as previously reported in REGARDS (PMID: 27792645) and CTS (PMID: 29723004) papers.
- Furthermore, we now cite Gerritsen et al. (PMID: 32427758). Thank you for that citation. See lines 84-87 on page 4.

Be aware that you are using several fonts within one paragraph!

- Thank you for this comment. We have made sure to format the entire paper consistently.

**Methods**

The description of the classification of whether a participant was a caregiver or not is confusing. Maybe this is due to the again alternating fonts, but possibly also because it seems as if you are repeating your self. Please rephrase lines 176-188.

- Thank you for this comment. We have addressed the alternating fonts issue and have rephrased the classification of caregiving and control status. See lines 142-167 on pages 6-7.

Statistical analysis- Why include all variables that were used to match the control participants as covariates as well? The good thing of using a matched design is that you would not have to adjust for potential confounders as theoretically your cases and controls are highly comparable on those factors.

- We thank the reviewer for this comment. We have made the decision to include the variables as covariates in the model even though they were generally matched. Matching gives good control over possible confounding, but including covariates, even if groups are matched, also accounts for any variability in the outcome (change in TL) that would otherwise be considered error variability.
- Additionally, including covariates in the model can increase precision in the effect estimate, as well as reduce the bias due to residual imbalance making the estimate “doubly robust” (<https://cran.r-project.org/web/packages/MatchIt/vignettes/estimating-effects.html>).

Figure 1 does not add to the results already shown in table 2. It also does not add new results to already existing data: we know there is a significant relation between telomere length at baseline and followup, similarly as there is with change between follow-up and baseline telomere length. The regression line is not interesting, as it is a general line and there are no separate lines for the caregivers and the controls. Which would also not lead to anything of interest, as there was no effect of caregiver status on telomere length. So I would suggest to delete this figure.

- The authors thank the reviewer for this comment. We have deleted Figure 1, as there really wasn’t any valuable information that is provided past the general quality control (assuring the relationship between telomere length at the separate visits). This information is also highlighted within the supplementary table 2 and therefore is not needed as a visual representation.

**Discussion**

In the Discussion a large part is described about what other studies found with health-related outcomes. This is interesting, but I am missing how the authors think that health outcomes are related to telomere shortening. The link between health outcomes, stress and telomere length is currently missing, which in my view is crucial to make this relevant for the current study.

- The authors thank the reviewer for this comment. We too believe that this link is crucial to the current study and apologize for its original omission. We have added a more complete analysis from lines 315-323 on page 14. We have included the following lines specifically: “It has been hypothesized that a major stress exposure, such as caregiving, can lead to chronically high levels of perceived stress and/or psychological stress arousal leading to a reduction in telomere maintenance and thus a reduction in TL [21, 45]. Previous studies have explored the relationship between TL and health, and have found shorter TL to be a risk factor for diseases of aging such as cancer [46] and cardiometabolic dysfunction [21, 47]. In the current study, while we observed differences in the degree of perceived stress between caregivers and controls, we did not find any association with perceived stress or the caregiving transition and changes in TL among all ~400 participants or in the caregiving subpopulation.”

The scores on the perceived stress scale were very low, also for the caregivers. Please elaborate on how this could be and how this may have affected your results. Was this the same in other caregiver studies?

- The authors thank the reviewer for this comment. As we have mentioned above, our study is not a clinical study and the participants, for the most part, do not report being overly stressed at either visit. We have included additional methodologies on the 4-item short form of the PSS questionnaire, as well as its validity and how it has previously been used in REGARDS publications (see page 8, lines 183-190). Furthermore, we have included additional sensitivity analyses using PSS as a categorical variable, dividing our subset into tertiles, as previously described, and evaluating the change in telomere length for each tertile across both caregivers and controls and within the caregivers only (supplementary table 3). We did not observe any difference in mean telomere length across perceived stress tertile. In the present study, we did observe a statistically significant difference among perceived stress categories between caregivers and controls, where controls tended to have higher frequency of low- to moderate-PSS and caregivers had a greater frequency of higher-PSS (Table 1).
- It’s difficult to compare stress levels in our study to other studies due to differences in the stress scale data collected. In Epel et al., 2010 (PMID:20018236), caregivers recruited from community and service centers for caregivers (e.g. support groups, neurology clinics, adult daycares) reported higher levels of perceived life stress (measured using the 10-item scale ranging from 0-40). The mean (SE) PSS for a caregiver was 19.1 (1.1) versus 9.9 (1.3) in controls. This difference in observed PSS could be due to population-based versus convenience-based recruitment of caregivers.
